# Gold, silver or bronze: circadian variation strongly affects performance in Olympic athletes

**DOI:** 10.1038/s41598-020-72573-8

**Published:** 2020-10-08

**Authors:** R. Lok, G. Zerbini, M. C. M. Gordijn, D. G. M. Beersma, R. A. Hut

**Affiliations:** 1 Chronobiology Unit, Groningen Institute for Evolutionary Life Sciences, University of Groningen, PO Box 11103, 9700CC Groningen, The Netherlands; 2 University of Groningen, Campus Fryslân, Wirdumerdijk 34, 8911 CE Leeuwarden, The Netherlands; 3 Department of Medical Psychology and Sociology, University of Augsburg, Augsburg, Germany; 4 Chrono@Work B.V, Frieschestraatsweg 213, 9743 AD Groningen, The Netherlands; 5 Present Address: Department of Psychiatry and Behavioral Sciences, Stanford University, 401 Quarry Road, Palo Alto, CA 94305 USA

**Keywords:** Neuroscience, Physiology

## Abstract

The circadian system affects physiological, psychological, and molecular mechanisms in the body, resulting in varying physical performance over the day. The timing and relative size of these effects are important for optimizing sport performance. In this study, Olympic swim times (from 2004 to 2016) were used to determine time-of-day and circadian effects under maximal motivational conditions. Data of athletes who made it to the finals (N = 144, 72 female) were included and normalized on individual levels based on the average swim times over race types (heat, semifinal, and final) per individual for each stroke, distance and Olympic venue. Normalized swim times were analyzed with a linear mixed model and a sine fitted model. Swim performance was better during finals as compared to semi-finals and heats. Performance was strongly affected by time-of-day, showing fastest swim times in the late afternoon around 17:12 h, indicating 0.32% improved performance relative to 08:00 h. This study reveals clear effects of time-of-day on physical performance in Olympic athletes. The time-of-day effect is large, and exceeds the time difference between gold and silver medal in 40%, silver and bronze medal in 64%, and bronze or no medal in 61% of the finals.

## Introduction

Circadian rhythms, regulated by the Suprachiasmatic Nucleus (SCN), influence many aspects associated with physiological performance, such as muscle strength^1^, and muscle flexibility^2^, in addition to perceptual and cognitive aspects of performance^3^. Strong correlations between physical performance and (circadian) variation in core body temperature (CBT) have been assessed, with optimal physical performance coinciding with the peak in CBT in the early evening^4–7^. Passive heating of muscles improves physical performance, indicating that either thermoregulation^8^, muscle temperature^9,10^ or both influence physical performance, although other factors (such as insulin, cortisol, total and free testosterone, oxygen uptake, glucose, growth hormone, norepinephrine^11^, and melatonin release^8^) also play a role^12^. Depending on the type of exercise (e.g. short-term or long-term, aerobic or anaerobic, individual sport or team sport), the involvement of psychological aspects (e.g. motivation, concentration), external conditions (e.g. cold vs. hot environments), and time-of-day effects on physical performance vary^8,13,14^. Additionally, variations in chronotype (which describes an individual’s biological optimal timing for activity and sleep), relate to substantial variations in peak performance time^15,16^.

Studies investigating these effects on elite athletes during high-level competitions are scarce. The Olympic venues are leading international sporting events, with thousands of athletes from around the world. The country selected to host the Olympics, sometimes adjusts race times to accommodate prime-broadcasting times in other continents. As a result, athletes are often required to perform at different, and sometimes unusual, times of day. This variation can be used to analyze time-of-day effects on physical performance in professional, extremely motivated male and female athletes. The goal of this study was to determine if Olympic athletes are affected by circadian fluctuations in physical performance, by analyzing Olympic swim data from the Games of Athens (2004), Beijing (2008), London (2012) and Rio de Janeiro (2016). Swimming requires minimal aiding materials (such as bikes, shoes) that could induce variation within and between athletes, and water temperature is mandated to vary within 25 to 28 degrees Celsius (by the Fédération internationale de natation), which forces water temperatures to be within the same range between Olympic venues. Swimming is therefore less likely to be influenced by confounding environmental effects (such as environmental temperature, humidity, wind etc.), and, of all sports types, we therefore expect that Olympic swim performance may reveal a very clean signal of daily variation in physical (e.g. muscle) performance. Our results can lead to strategies to significantly improve individual swimming performance.

## Results

### Effects of race type and time-of-day

**Figure 1:**
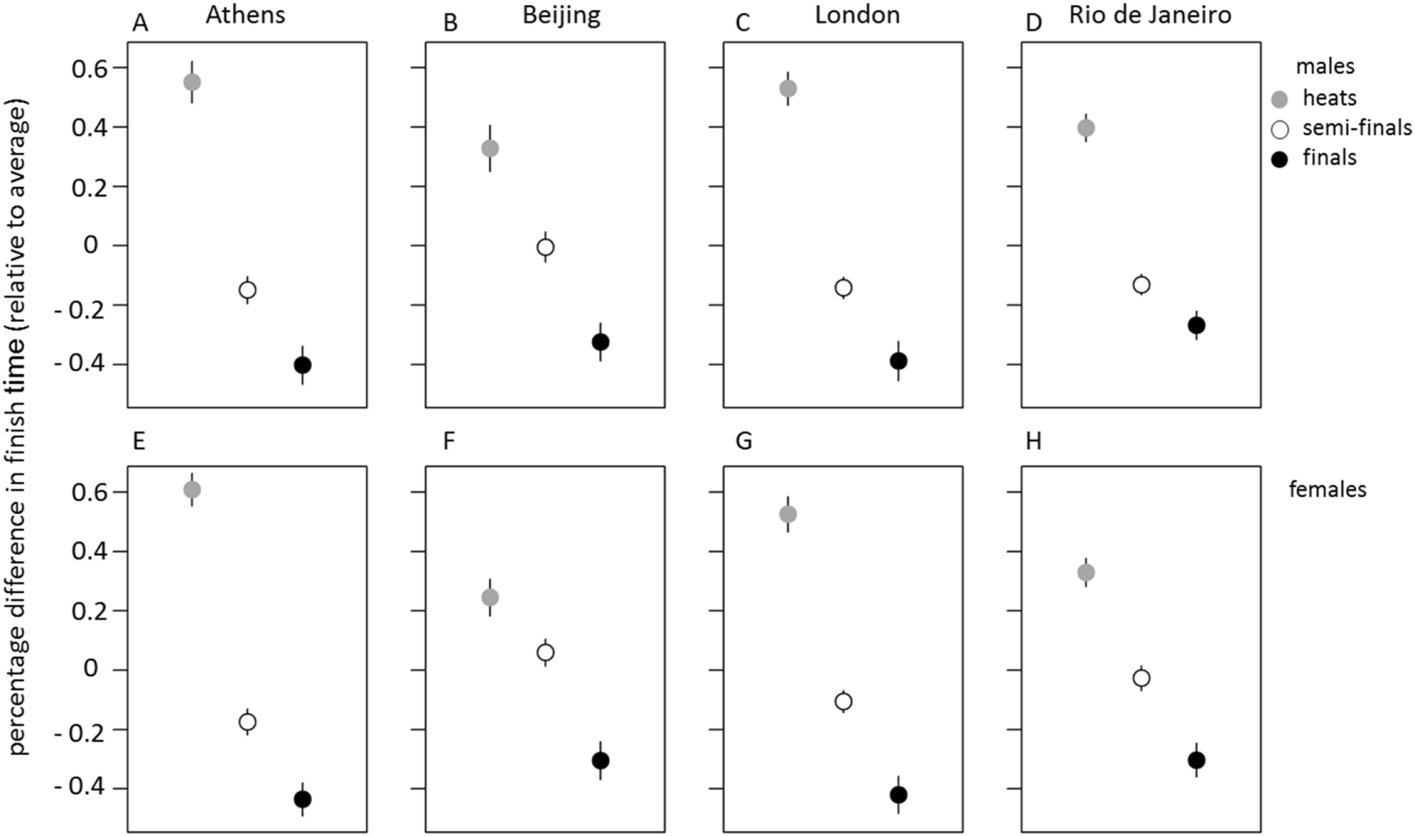
Normalized swim scores of Olympic venues in Athens (**A**,**E**), Beijing (**B**,**F**), London (**C**,**G**) and Rio de Janeiro (**D**,**H**). Data is plotted as mean ± standard error of the mean, with grey dots representing swim times collected during heats, and white and black dots representing swim data collected during semi-finals and finals, respectively. Top row (**A**–**D**) indicates male finish times, while bottom row (**E**–**H**) depicts finish times of female athletes.

Data analysis on within subject normalized data revealed that race type significantly affected swim performance (Fig. 1). Heats were 0.5% slower than semi-finals, which in turn were 0.2% slower than finals, in females (F_2,850_ = 225.05, p < 2 × 10^−16^) and males (F_2,850_ = 220.07, p < 2 × 10^–16^). There was a significant interaction between Olympic venue and race type (F_9,850_ = 4.71, p < 1 × 10^–6^) for females and males (F_9,850_ = 1.97, p = 0.039), suggesting that performance differences between race types varied between Olympic venue locations. The percentage difference in swim times between heats and finals in Beijing (0.60%, average of males and females) was much smaller than in Athens and in London (0.99% and 0.93% respectively). A major difference between those Games is that the finals in Beijing were held at about the time of the heats in London and Athens, while the heats in Beijing were held at about the time of the finals in London and Athens. This scheduling difference is an interesting opportunity to disentangle motivation (faster swim times in the finals) from possible time-of-day effects. In fact, if time-of-day did not play a role, one would expect the same percentage difference in swim times between heats and finals in Beijing as in Athens and London. To test for time-of-day effects we fitted a sine model.

The linear mixed model (in which normalized finish times were explained by race type, Game venue and time-of-day) indicated significant effects of race type (F_2,1715_ = 440.26, p < 1 × 10^−15^), Olympic venue (F_3,1715_ = 0.02, p = 0.05), and time-of-day (period =24 h; F_2,1715_ = 11.94, p < 1 × 10^−5^).

**Figure 2:**
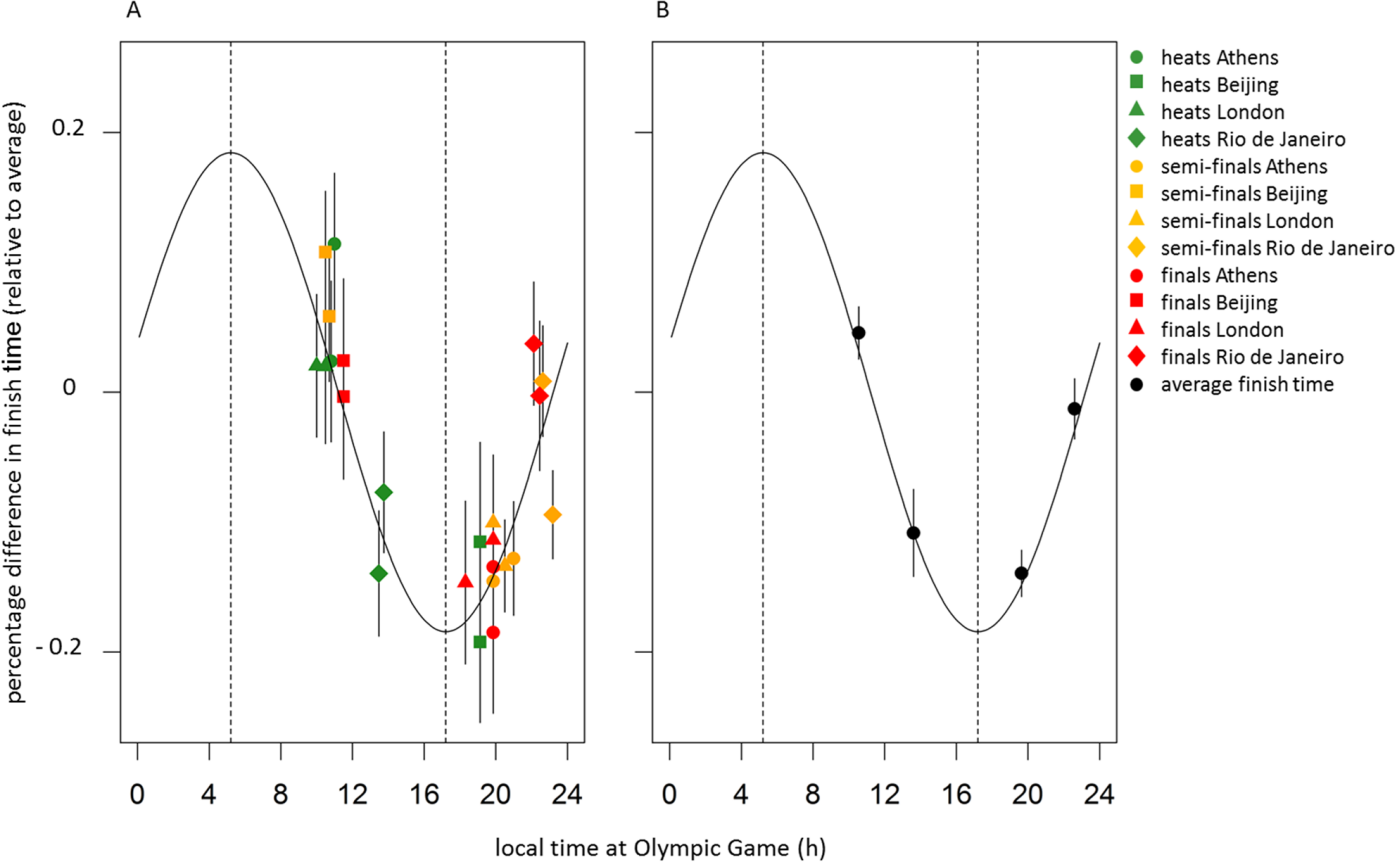
Olympic swim performance depends on time-of-day. Residual variation of individually normalized data of heats, semi-finals and finals (corrected for intercept, type of race, Olympic venue, and individual differences, as quantified by a linear mixed model), was fitted by a 24-h period sine function and plotted against local time at the Olympic venue location (h). Data represent mean ± SEM. (**A**) Data collected during heats (green), semi-finals (orange) and finals (red). (**B**) Black dots indicate average finish times in 3-h bins (**B**). Sine fit (period = 24 h, black curve) describing variation in swim performance over the day, indicates worst performance in the early morning and best performance in the late afternoon (dotted lines).

The sine fitted model (Fig. 2) predicted that swim performance would be worst in the early morning (5:12 h), and best in the late afternoon (17:12 h). There was no significant difference depending on sex, therefore the same sine wave was plotted for both males and females (see Supplemental Digital Content, Fig. S1 for the data plotted separately for males and females).

### The relative magnitude of time-of-day effects

The amplitude range of the fitted sine wave representing the effects of time-of-day is 0.37% (peak-to-trough distance, Fig. 2). In 40% of the finals, this time-of-day effect was larger than the time difference between gold or silver medal finishing times (Supplemental Digital Content, Table S1). Moreover, time-of-day effects exceed the time difference between the silver and bronze medal in 64% of the finals, and the time difference between bronze or fourth place in 61% of the finals (Supplemental Digital Content, Tables S2, S3).

## Discussion

The current analysis reveals that Olympic athletes always perform better in finals compared to semi-finals and heats (probably due to motivational differences) and that physical performance assessed in Olympic athletes was significantly affected by time-of-day. Best performance was determined in the late afternoon. This indicates that, despite of elaborate training schedules ranging from morning to evening hours, time-of-day still affects professional athletes’ performance. Physical performance is therefore not determined by training only, but also by the endogenous circadian system. Some studies indicate that physical performance at a specific time-of-day can improve after repeatedly training at that time-of-day, suggesting that the trough observed in morning performance can be partially counteracted^17^. This time-of-day effect may depend on CBT levels. On one hand, cold water immersion in the afternoon decreases CBT levels to morning levels, as well as it decreases evening- to morning performance levels. On the other hand, passive increase (i.e. variation in environmental temperature) in CBT rescues impaired morning performance^8,12,18^, similar to hot water immersion^12^ and active warm-up^19,20^, that also improve time-of-day related decrements in performance, by increasing CBT or muscle temperature levels.

Internal clock time also influences physical performance, causing early chronotypes to perform best around mid-day, intermediate chronotypes around mid-afternoon, and late chronotypes in the evening^16^. It is therefore possible that morning races benefit early types, while evening races benefit later types. Swim training times are often scheduled in the early morning, therefore a selection bias towards earlier chronotypes can exist, as has been determined in other sports^21^. Later chronotypes are also associated with more diurnal variation in performance, which might cause an additional selection pressure towards earlier chronotypes, particularly in Olympic athletes^16^. The optimal performance peak in finish times analyzed here occurs relatively early compared to the peak in CBT timing^1,22,23^, which may indicate an over-representation of early chronotypes (with earlier CBT peak times) among Olympic swimmers.

Various circadian rhythms in the body may contribute to time-of-day variation in physical performance. Limb movement speed and muscle strength depend on time-of-day^1^, as well as muscle flexibility and grip strength^2^. Improved performance coincides with lower levels of insulin, cortisol, total and free testosterone, and higher oxygen uptake, aerobic mechanical power output, metabolic rate and concentrations of glucose and growth hormone^11^. Moreover, factors such as sleep duration, -quality and sleep inertia influence performance^24,25^. Here we could not collect data on sleep in athletes prior and during the Olympics and we can therefore not disentangle between circadian and homeostatic effects. The optimum in physical performance might therefore depend on a complex combination of mental performance, time awake, circadian rhythm in muscle cells and mitochondrial oxygen consumption.

The current analysis only includes individuals who made it to the finals, which may have induced a bias to more successful athletes. Athletes that suffer more from time-of-day effects might have been excluded because they did not reach the finals, resulting in an underestimation of the time-of-day effect. Shorter recovery time is also associated with impaired physical performance^26,27^. In London and Athens, heats and semi-finals were scheduled approximately 9 h apart, while in Beijing, recovery time after heats was 13.5 h on average. Yet, differences in performance between heats and semi-finals are smaller in Beijing compared to other Olympic venues, suggesting that time-of-day effects counteracted beneficial effects of longer recovery time, including a night's sleep.

Our analysis concerns only swimmers and therefore generalization to other sports might be difficult. However, we chose to analyze specifically this sport because swimming requires minimal aiding materials (such as bikes or shoes) that could induce variation within and between athletes, while water temperature varies within a relatively narrow range, further minimizing confounding factors. In addition, swimming employs muscles in both arms and legs. Since there is no indication that muscle clocks differ over the body between arms and legs, we expect that this circadian effect on swim performance is actually reflecting a general variation over the day in muscle performance, and could therefore affect other sport performance in a similar manner.

In 40% of the races, the time-of-day effect is bigger than the difference between finishing first or second. Moreover, the time-of-day effect exceeds the time difference between silver and bronze in 64% of the finals, and the time difference between bronze or fourth place in 61% of the finals. In upcoming Olympic venues, swimmers and other athletes may have to perform at times of day that do not coincide with their circadian peak performance. Shifting peak performance to better match each race type is difficult, since heats and semi-finals for instance are often on the same day in the morning and in the afternoon/evening. Depending on one’s goal (reaching the semi-final or winning the final) athletes may consider to adjust their circadian system such that their peak performance better matches race timing accordingly.

## Methods

### Data collection

All data concerning participating athletes, swim schedules and pertinent finish times of Olympic venues of Athens, Beijing, London and Rio de Janeiro were obtained from https://www.olympic.org/ (publically accessible from official reports). Athens Olympic swim schedules were analyzed using Eastern Standard Time, Beijing swim schedules using China Standard Time, London swimming schedules using Greenwich Mean Time, and Rio de Janeiro using Brasilia Standard Time. Olympic swim contests exist of three race types: heats (varying number of competing athletes), from which the 16 fastest finish times can partake in semi-finals, after which the 8 highest ranked athletes participate in finals. To ensure a homogenous sample of athletes, only athletes that qualified for the finals were included, resulting in a total of 144 athletes (72 female) per Olympic venue. The breakdown of athletes per Olympic venue can be found in Fig. S2. Data of all four Olympic venues consisted of four different strokes in two or three distances, resulting in nine different combinations: backstroke (100 and 200 m), breaststroke (100 and 200 m), butterfly (100 and 200 m), and freestyle (50, 100, and 200 m). Both at the Olympic venue of Athens and Beijing, one finalist was disqualified (at the 200 m breaststroke and 100 m freestyle respectively), resulting in inclusion of 1722 data points in total for the current analysis.

### Data analysis (1)

#### Effects of race type and time-of-day

To exclude effects of novel training methods, techniques and equipment (e.g. shark suits used in Beijing^28^), data were normalized as follows: first the average swim time over race type (heats, semifinal and final), was calculated per individual, stroke, distance, and per Olympic venue; then the percentage difference between each race swim time (heat, semifinal and final) and the average swim time was calculated for each combination of stroke and distance. This normalization method allowed for inclusion of all available swim strokes and distances in a single linear mixed model. To assess differences between race type, normalized swim scores were plotted separately for heats, semi-finals and finals per Olympic venue.

### Data analysis (2)

#### Time-of-day

To accommodate differences in race timing (finals in the morning in Beijing, whereas held in the evening in Athens and London), we compared swim times between all four Olympics venues in a linear model (R-studio, version 1.0.136), with swim time (as calculated (1)) as dependent variable, and as independent variables type of race (heat, semifinal or final), Olympic venue location, and time-of-day (as a sine function). Subject identity was included as random effect, to control for between-subject variation. The data distribution was normal (the Shapiro–Wilk normality test (w = 0.979, p < 2.2^e−16^) justifying usage of the linear mixed model. To visualize time-of-day effects, the residual variation after subtraction of the components race type, Olympic venue, individual, and intercept of the linear mixed model from the normalized data was calculated. This residual variation was plotted against local time at Olympic venue (h) and the sine function that resulted from the linear mixed model was plotted through the data.

### Data analysis (3)

#### Effect size

The relative magnitude of the time-of-day effect was assessed by comparing it to the relative time difference between the first and the second place, which was calculated by dividing their time difference by second finishing time.

## Supplementary information

Supplementary Information

## Data Availability

Original data is publically accessible at https://www.olympic.org/. Analyzed data and R codes can be accessed by contacting the corresponding author. The complete dataset (original and analyzed) and R codes are also available at the data repository of the University of Groningen.
