# Supplementary material for: Gold, silver or bronze: circadian variation strongly affects performance in Olympic athletes
Source: Sci Rep. 2020 Oct 8;10:16088. doi: 10.1038/s41598-020-72573-8 (PMC7544825; doi:10.1038/s41598-020-72573-8)
Supplement: Supplementary file 1 — Supplementary Information [file 41598_2020_72573_MOESM1_ESM.pdf]

Supplemental Materials for

Gold, silver or bronze: circadian variation strongly affects performance in Olympic athletes

R. Lok, G. Zerbini, M.C.M. Gordijn, D.G.M. Beersma, R.A. Hut

Correspondence to: Renske Lok, Current address: University of Stanford, department of Psychiatry, 3801 Miranda Avenue, Palo Alto, California 94304, e-mail: rlok@stanford.edu

This PDF file includes:

Fig. S1 – S2

Table S1 – S4

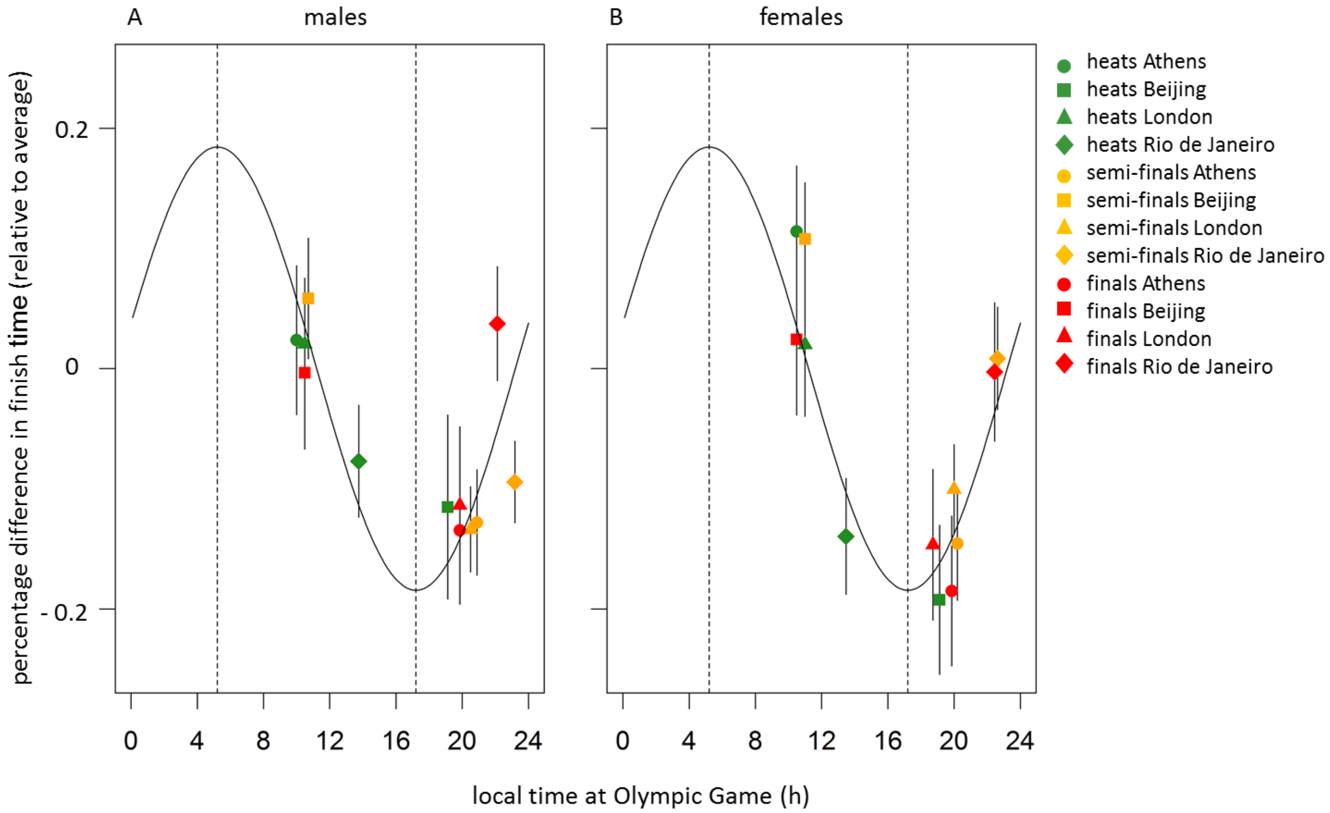

Fig. S1. Swim performance as a function of time of day in male and female athletes. Residual variation of individually normalized data of heats, semi-finals and finals (containing type of race, Olympic Game) plotted as percentage difference in finishing time, was fitted by a 24-h period sine function and plotted against local time at the Olympic Game location (h). Data represent mean ± SEM. Data collected during heats (green), semi-finals (orange) and finals (red) in male (A) and female (B) athletes. Sine fit (period=24 h, black curve) describing variation in swim performance over the day, indicates worst performance in the late morning and best performance in the late evening (dotted lines). Since normalized performance did not differ in phase and amplitude between genders ($F_{2,1719}$=0.84, p=0.53), the same significant sinusoid was fitted through the combined data set.

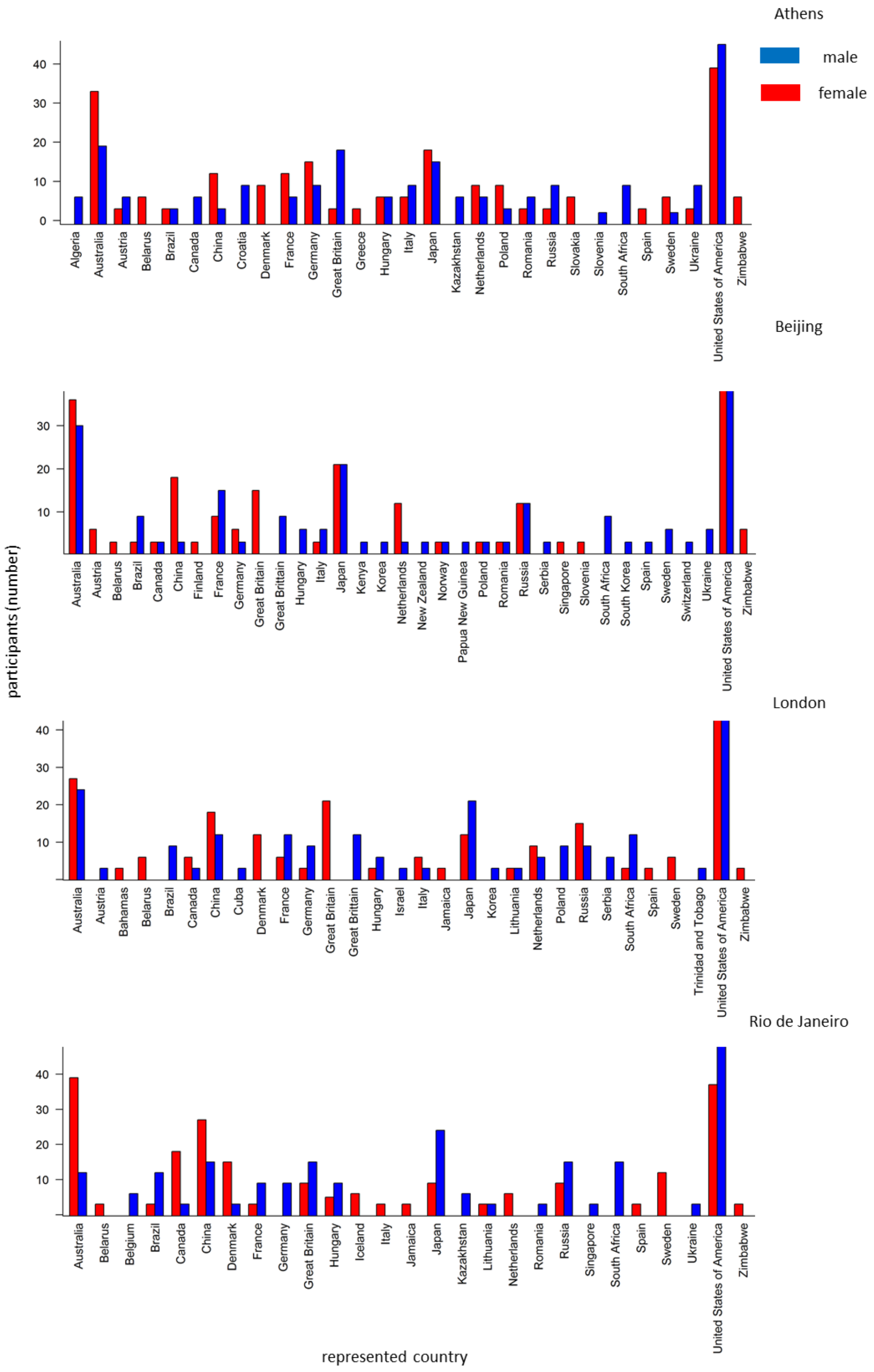

Figure S2: Breakdown of the represented countries at the Olympic Games of Athens, Beijing, London and Rio de Janeiro.

Table S1: Overview of first or second place finish times at the Olympic Games of Athens, Beijing, London and Rio de Janeiro. In 40% of the finals, the difference between finishing first or second is smaller than the variance predicted by time of day (including one joint first place).

Stroke	Distance	Olympic venue	Gender	First place finish time (s)	Second place finish time (s)	Difference (%)	Time of day effect (0.369%) bigger?
Backstroke	100	Athens	Female	60.37	60.50	0.215	Yes
Backstroke	100	Beijing	Female	58.96	59.19	0.389	No
Backstroke	100	London	Female	58.33	58.68	0.596	No
Backstroke	100	Rio de Janeiro	Female	58.45	58.75	0.511	No
Backstroke	100	Athens	Male	54.06	54.35	0.534	No
Backstroke	100	Beijing	Male	52.54	53.11	1.073	No
Backstroke	100	London	Male	52.16	52.92	1.436	No
Backstroke	100	Rio de Janeiro	Male	51.97	52.31	0.650	No
Backstroke	200	Athens	Female	129.19	129.72	0.409	No
Backstroke	200	Beijing	Female	125.24	126.23	0.784	No
Backstroke	200	London	Female	124.06	125.92	1.477	No
Backstroke	200	Rio de Janeiro	Female	125.99	126.05	0.048	Yes
Backstroke	200	Athens	Male	114.95	117.35	2.045	No
Backstroke	200	Beijing	Male	113.94	114.33	0.341	Yes
Backstroke	200	London	Male	113.41	113.78	0.325	Yes
Backstroke	200	Rio de Janeiro	Male	113.62	113.96	0.298	Yes
Breaststroke	100	Athens	Female	66.60	67.20	0.893	No
Breaststroke	100	Beijing	Female	65.17	66.73	2.338	No
Breaststroke	100	London	Female	65.47	65.55	0.122	Yes
Breaststroke	100	Rio de Janeiro	Female	64.93	65.50	0.870	No
Breaststroke	100	Athens	Male	60.08	60.25	0.118	Yes
Breaststroke	100	Beijing	Male	58.91	59.20	0.490	No
Breaststroke	100	London	Male	58.46	58.93	0.454	No
Breaststroke	100	Rio de Janeiro	Male	57.13	58.69	2.658	No
Breaststroke	200	Athens	Female	143.37	143.60	0.160	Yes
Breaststroke	200	Beijing	Female	140.22	142.05	1.288	No
Breaststroke	200	London	Female	139.59	140.72	0.803	No
Breaststroke	200	Rio de Janeiro	Female	140.30	141.97	1.176	No
Breaststroke	200	Athens	Male	129.44	130.88	2.658	No
Breaststroke	200	Beijing	Male	127.64	128.88	0.798	No
Breaststroke	200	London	Male	127.28	127.43	0.020	Yes
Breaststroke	200	Rio de Janeiro	Male	127.46	127.53	0.055	Yes
Butterfly	100	Athens	Female	57.72	57.84	0.207	Yes
Butterfly	100	Beijing	Female	56.73	57.10	0.648	No
Butterfly	100	London	Female	55.98	56.87	1.565	No

Butterfly	100	Rio de Janeiro	Female	55.48	56.46	1.736	No
Butterfly	100	Athens	Male	51.25	51.29	0.078	Yes
Butterfly	100	Beijing	Male	50.58	50.59	0.020	Yes
Butterfly	100	London	Male	51.21	51.44	0.447	No
Butterfly	100	Rio de Janeiro	Male	50.39	51.14	1.467	No
Butterfly	200	Athens	Female	126.05	126.36	0.245	Yes
Butterfly	200	Beijing	Female	124.18	124.72	0.433	No
Butterfly	200	London	Female	124.06	125.25	0.950	No
Butterfly	200	Rio de Janeiro	Female	124.85	124.88	0.024	Yes
Butterfly	200	Athens	Male	114.04	114.56	0.454	No
Butterfly	200	Beijing	Male	112.03	112.70	0.594	No
Butterfly	200	London	Male	112.96	113.01	0.044	Yes
Butterfly	200	Rio de Janeiro	Male	113.36	113.40	0.035	Yes
Freestyle	50	Athens	Female	24.58	24.89	1.245	No
Freestyle	50	Beijing	Female	24.06	24.07	0.042	Yes
Freestyle	50	London	Female	24.05	24.28	0.947	No
Freestyle	50	Rio de Janeiro	Female	24.07	24.09	0.083	Yes
Freestyle	50	Athens	Male	21.93	21.94	0.046	Yes
Freestyle	50	Beijing	Male	21.30	21.45	0.699	No
Freestyle	50	London	Male	21.34	21.54	0.929	No
Freestyle	50	Rio de Janeiro	Male	21.40	21.41	0.047	Yes
Freestyle	100	Athens	Female	53.84	54.16	0.591	No
Freestyle	100	Beijing	Female	53.12	53.16	0.075	Yes
Freestyle	100	London	Female	53.00	53.38	0.712	No
Freestyle	100	Rio de Janeiro	Female	52.70	52.70	0.000	Yes
Freestyle	100	Athens	Male	48.17	48.23	0.124	Yes
Freestyle	100	Beijing	Male	47.21	47.32	0.232	Yes
Freestyle	100	London	Male	47.52	47.53	0.021	Yes
Freestyle	100	Rio de Janeiro	Male	47.58	47.80	0.460	No
Freestyle	200	Athens	Female	118.03	118.22	0.161	Yes
Freestyle	200	Beijing	Female	114.80	115.00	0.174	Yes
Freestyle	200	London	Female	113.61	115.58	1.704	No
Freestyle	200	Rio de Janeiro	Female	113.73	114.08	0.307	Yes
Freestyle	200	Athens	Male	104.71	105.23	0.494	No
Freestyle	200	Beijing	Male	102.96	104.85	1.803	No
Freestyle	200	London	Male	103.14	104.93	1.706	No
Freestyle	200	Rio de Janeiro	Male	104.65	105.20	0.523	No

Table S2: Overview of second (silver medal) or third place (bronze medal) finish times at the Olympic Games of Athens, Beijing, London and Rio de Janeiro. In 64% of the finals, the difference between finishing second or third is smaller than the variance predicted by time of day (including three joint second places).

Stroke	Distance	Olympic venue	Gender	Second place finish time (s)	Third place finish time (s)	Difference (%)	Time of day effect (0.369%) bigger?
Backstroke	100	Athens	Female	60.50	60.88	0.624	No
Backstroke	100	Beijing	Female	59.19	59.34	0.253	Yes
Backstroke	100	London	Female	58.68	58.83	0.255	Yes
Backstroke	100	Rio de Janeiro	Female	58.75	58.76	0.017	Yes
Backstroke	100	Athens	Male	54.35	54.36	0.018	Yes
Backstroke	100	Beijing	Male	53.11	53.18	0.132	Yes
Backstroke	100	London	Male	52.92	52.97	0.094	Yes
Backstroke	100	Rio de Janeiro	Male	52.31	52.40	0.172	Yes
Backstroke	200	Athens	Female	129.72	129.88	0.123	Yes
Backstroke	200	Beijing	Female	126.23	127.13	0.708	No
Backstroke	200	London	Female	125.92	126.55	0.498	No
Backstroke	200	Rio de Janeiro	Female	126.05	127.54	1.168	No
Backstroke	200	Athens	Male	117.35	117.56	0.179	Yes
Backstroke	200	Beijing	Male	114.33	114.93	0.522	No
Backstroke	200	London	Male	113.78	113.94	0.140	Yes
Backstroke	200	Rio de Janeiro	Male	113.96	113.97	0.009	Yes
Breaststroke	100	Athens	Female	67.15	67.16	0.015	Yes
Breaststroke	100	Beijing	Female	66.73	67.34	0.906	No
Breaststroke	100	London	Female	65.55	66.46	1.369	No
Breaststroke	100	Rio de Janeiro	Female	65.50	65.69	0.289	Yes
Breaststroke	100	Athens	Male	60.25	60.88	1.035	No
Breaststroke	100	Beijing	Male	59.20	59.37	0.286	Yes
Breaststroke	100	London	Male	58.93	59.49	0.941	No
Breaststroke	100	Rio de Janeiro	Male	58.69	58.87	0.306	Yes
Breaststroke	200	Athens	Female	143.6	145.82	1.522	No
Breaststroke	200	Beijing	Female	142.05	143.02	0.678	No
Breaststroke	200	London	Female	140.72	140.92	0.142	Yes
Breaststroke	200	Rio de Janeiro	Female	141.97	142.28	0.218	Yes
Breaststroke	200	Athens	Male	130.80	130.87	0.053	Yes
Breaststroke	200	Beijing	Male	128.88	128.94	0.047	Yes
Breaststroke	200	London	Male	127.43	128.29	0.670	No
Breaststroke	200	Rio de Janeiro	Male	127.53	127.7	0.133	Yes
Butterfly	100	Athens	Female	57.84	57.99	0.259	Yes
Butterfly	100	Beijing	Female	57.10	57.25	0.262	Yes
Butterfly	100	London	Female	56.87	56.94	0.123	Yes

Butterfly	100	Rio de Janeiro	Female	56.46	56.63	0.300	Yes
Butterfly	100	Athens	Male	51.29	51.36	0.136	Yes
Butterfly	100	Beijing	Male	50.59	51.12	1.037	No
Butterfly	100	London	Male	51.44	51.44	0.000	Yes
Butterfly	100	Rio de Janeiro	Male	51.14	51.14	0.000	Yes
Butterfly	200	Athens	Female	126.36	128.04	1.312	No
Butterfly	200	Beijing	Female	124.70	126.26	1.236	No
Butterfly	200	London	Female	125.25	125.48	0.183	Yes
Butterfly	200	Rio de Janeiro	Female	124.88	125.2	0.256	Yes
Butterfly	200	Athens	Male	114.56	115.52	0.831	No
Butterfly	200	Beijing	Male	112.70	112.97	0.239	Yes
Butterfly	200	London	Male	113.01	113.21	0.177	Yes
Butterfly	200	Rio de Janeiro	Male	113.4	113.62	0.194	Yes
Freestyle	50	Athens	Female	24.89	24.91	0.080	Yes
Freestyle	50	Beijing	Female	24.07	24.17	0.414	No
Freestyle	50	London	Female	24.28	24.39	0.451	No
Freestyle	50	Rio de Janeiro	Female	24.09	24.11	0.083	Yes
Freestyle	50	Athens	Male	21.94	22.02	0.363	Yes
Freestyle	50	Beijing	Male	21.45	21.49	0.186	Yes
Freestyle	50	London	Male	21.54	21.59	0.232	Yes
Freestyle	50	Rio de Janeiro	Male	21.41	21.49	0.372	No
Freestyle	100	Athens	Female	54.16	54.4	0.441	No
Freestyle	100	Beijing	Female	53.16	53.39	0.431	No
Freestyle	100	London	Female	53.38	53.44	0.112	Yes
Freestyle	100	Rio de Janeiro	Female	52.70	52.99	0.547	No
Freestyle	100	Athens	Male	48.23	48.56	0.680	No
Freestyle	100	Beijing	Male	47.32	47.67	0.734	No
Freestyle	100	London	Male	47.53	47.80	0.565	No
Freestyle	100	Rio de Janeiro	Male	47.80	47.85	0.104	Yes
Freestyle	200	Athens	Female	118.22	118.45	0.194	Yes
Freestyle	200	Beijing	Female	114.97	115.04	0.061	Yes
Freestyle	200	London	Female	115.58	115.81	0.199	Yes
Freestyle	200	Rio de Janeiro	Female	114.08	114.92	0.731	No
Freestyle	200	Athens	Male	105.23	105.32	0.085	Yes
Freestyle	200	Beijing	Male	104.85	105.14	0.276	Yes
Freestyle	200	London	Male	104.93	104.93	0.000	Yes
Freestyle	200	Rio de Janeiro	Male	105.20	105.23	0.029	Yes

Table S3: Overview of third (bronze medal) or fourth place (no medal) finish times at the Olympic Games of Athens, Beijing, London and Rio de Janeiro. In 61% of the finals, the difference between finishing third or fourth is smaller than the variance predicted by time of day (including five joint third places).

Stroke	Distance	Olympic venue	Gender	Third place finish time (s)	Fourth place finish time (s)	Difference (%)	Time of day effect (0.369%) bigger?
Backstroke	100	Athens	Female	60.88	61.05	0.278	Yes
Backstroke	100	Beijing	Female	59.34	59.38	0.067	Yes
Backstroke	100	London	Female	58.83	59.00	0.288	Yes
Backstroke	100	Rio de Janeiro	Female	58.76	58.76	0.000	Yes
Backstroke	100	Athens	Male	54.36	54.38	0.037	Yes
Backstroke	100	Beijing	Male	53.18	53.18	0.000	Yes
Backstroke	100	London	Male	52.97	53.08	0.207	Yes
Backstroke	100	Rio de Janeiro	Male	52.4	52.43	0.057	Yes
Backstroke	200	Athens	Female	129.88	129.88	0.000	Yes
Backstroke	200	Beijing	Female	127.13	127.88	0.586	No
Backstroke	200	London	Female	126.55	127.26	0.558	No
Backstroke	200	Rio de Janeiro	Female	127.54	127.89	0.274	Yes
Backstroke	200	Athens	Male	117.56	117.76	0.170	Yes
Backstroke	200	Beijing	Male	114.93	115.49	0.485	No
Backstroke	200	London	Male	113.94	115.59	1.427	No
Backstroke	200	Rio de Janeiro	Male	113.97	115.16	1.033	No
Breaststroke	100	Athens	Female	67.16	67.44	0.415	No
Breaststroke	100	Beijing	Female	67.34	67.43	0.133	Yes
Breaststroke	100	London	Female	66.46	66.93	0.702	No
Breaststroke	100	Rio de Janeiro	Female	65.69	66.37	1.025	No
Breaststroke	100	Athens	Male	60.88	61.17	0.474	No
Breaststroke	100	Beijing	Male	59.37	59.57	0.336	Yes
Breaststroke	100	London	Male	59.49	59.53	0.067	Yes
Breaststroke	100	Rio de Janeiro	Male	58.87	59.22	0.591	No
Breaststroke	200	Athens	Female	145.82	145.87	0.034	Yes
Breaststroke	200	Beijing	Female	143.02	143.24	0.154	Yes
Breaststroke	200	London	Female	140.92	141.65	0.515	No
Breaststroke	200	Rio de Janeiro	Female	142.28	142.34	0.042	Yes
Breaststroke	200	Athens	Male	130.87	131.2	0.252	Yes
Breaststroke	200	Beijing	Male	128.94	129.03	0.070	Yes
Breaststroke	200	London	Male	128.29	128.35	0.047	Yes
Breaststroke	200	Rio de Janeiro	Male	127.7	127.78	0.063	Yes
Butterfly	100	Athens	Female	57.99	58.22	0.395	No
Butterfly	100	Beijing	Female	57.25	57.84	1.020	No
Butterfly	100	London	Female	56.94	57.17	0.402	No

Butterfly	100	Rio de Janeiro	Female	56.63	56.76	0.229	Yes
Butterfly	100	Athens	Male	51.36	52.27	1.741	No
Butterfly	100	Beijing	Male	51.12	51.13	0.020	Yes
Butterfly	100	London	Male	51.44	51.81	0.714	No
Butterfly	100	Rio de Janeiro	Male	51.14	51.26	0.234	Yes
Butterfly	200	Athens	Female	128.04	128.18	0.109	Yes
Butterfly	200	Beijing	Female	126.26	127.02	0.598	No
Butterfly	200	London	Female	125.48	125.78	0.239	Yes
Butterfly	200	Rio de Janeiro	Female	125.2	125.9	0.556	No
Butterfly	200	Athens	Male	115.52	116.00	0.414	No
Butterfly	200	Beijing	Male	112.97	114.35	1.207	No
Butterfly	200	London	Male	113.21	114.35	0.997	No
Butterfly	200	Rio de Janeiro	Male	113.62	114.06	0.386	No
Freestyle	50	Athens	Female	24.91	24.93	0.080	Yes
Freestyle	50	Beijing	Female	24.17	24.25	0.330	Yes
Freestyle	50	London	Female	24.39	24.46	0.286	Yes
Freestyle	50	Rio de Janeiro	Female	24.11	24.13	0.083	Yes
Freestyle	50	Athens	Male	22.02	22.08	0.272	Yes
Freestyle	50	Beijing	Male	21.49	21.62	0.601	No
Freestyle	50	London	Male	21.59	21.61	0.093	Yes
Freestyle	50	Rio de Janeiro	Male	21.49	21.68	0.876	No
Freestyle	100	Athens	Female	54.4	54.5	0.183	Yes
Freestyle	100	Beijing	Female	53.39	53.97	1.075	No
Freestyle	100	London	Female	53.44	53.47	0.056	Yes
Freestyle	100	Rio de Janeiro	Female	52.99	53.04	0.094	Yes
Freestyle	100	Athens	Male	48.56	48.63	0.144	Yes
Freestyle	100	Beijing	Male	47.67	47.67	0.000	Yes
Freestyle	100	London	Male	47.8	47.84	0.084	Yes
Freestyle	100	Rio de Janeiro	Male	47.85	47.88	0.063	Yes
Freestyle	200	Athens	Female	118.45	118.62	0.143	Yes
Freestyle	200	Beijing	Female	115.05	115.78	0.631	No
Freestyle	200	London	Female	115.81	115.82	0.009	Yes
Freestyle	200	Rio de Janeiro	Female	114.92	115.18	0.226	Yes
Freestyle	200	Athens	Male	105.32	106.13	0.763	No
Freestyle	200	Beijing	Male	105.14	105.97	0.783	No
Freestyle	200	London	Male	104.93	105.04	0.105	Yes
Freestyle	200	Rio de Janeiro	Male	105.23	105.49	0.246	Yes

Table S4: Selected example data explaining the concept of within subject normalization as employed in the current analysis. The average swim time over race type (heats, semifinal and final), was calculated per individual, stroke, distance, and per Olympic venue (**Average over all race types)**; then the percentage difference between each race swim time (heat, semifinal and final) and the average swim time was calculated for each combination of stroke and distance, resulting in the **Individually normalized score** column. A linear mixed model, with individually normalized scores as dependent variable and type of race (heat, semifinal or final), Olympic venue, and start time (h) as independent variables, and subject identity as random effect was employed. The residuals of this model result in the column **Model residuals**.

Subject identity	Olympic venue	Stroke	Distance (m)	Race type	Start time (h)	Finish time (s)	Average over all race types	Individually normalized score	Model residuals
1	Athens	Butterfly	100	heat	10.5	52.05	51.71667	1.006445	0.002024
1	Athens	Butterfly	100	semi-final	21.3	51.74	51.71667	1.000451	0.001274
1	Athens	Butterfly	100	final	22.6	51.36	51.71667	0.993103	-0.00327
2	Beijing	Breaststroke	100	heat	20.1	59.41	59.25667	1.002588	-0.00184
2	Beijing	Breaststroke	100	semi-final	10.0	59.16	59.25667	0.998369	-0.00082
2	Beijing	Breaststroke	100	final	11.5	59.20	59.25667	0.999044	0.002661
2	Beijing	Breaststroke	200	heat	21.3	128.68	129.21	0.995898	-0.00853
2	Beijing	Breaststroke	200	semi-final	9.6	129.73	129.21	1.004024	0.004838
2	Beijing	Breaststroke	200	final	10.8	129.22	129.21	1.000077	0.003695
3	London	Backstroke	200	heat	11.5	116.36	115.2333	1.009777	0.005346
3	London	Backstroke	200	semi-final	19.8	115.40	115.2333	1.001446	0.00226
3	London	Backstroke	200	final	20.5	113.94	115.2333	0.988776	-0.00761
4	Rio de Janeiro	Freestyle	50	heat	8.6	24.26	24.26667	0.999725	-0.00471
4	Rio de Janeiro	Freestyle	50	semi-final	19.4	24.41	24.26667	1.005907	0.00672
4	Rio de Janeiro	Freestyle	50	final	21.6	24.13	24.26667	0.994368	-0.00201
1	Rio de Janeiro	Butterfly	100	heat	10.5	51.78	51.51667	1.005112	0.00068
1	Rio de Janeiro	Butterfly	100	semi-final	20.8	51.51	51.51667	0.999871	0.000684
1	Rio de Janeiro	Butterfly	100	final	22.4	51.26	51.51667	0.995018	-0.00136
